# Facilitating quality improvement in primary healthcare using performance feedback and action planning

**DOI:** 10.1186/1472-6963-14-S2-P132

**Published:** 2014-07-07

**Authors:** Brigitte Vachon, Michel Camirand, Jean Rodrigue, Louise Quesnel, Jeremy Grimshaw

**Affiliations:** 1 School of Rehabilitation, Université de Montréal, Montreal, Quebec, Canada; 2 CSSS LaPommeraie, Cowansville, Quebec, Canada; 3 Agence de la Sante de la Monteregie, Longueuil, Quebec, Canada; 4 Ottawa Hospital Research Institute, Ottawa, Ontario, Canada

## Background

Optimizing primary healthcare requires changes at the system level, including professionals working together using quality improvement strategies, and accessing resources and support to implement these changes. Our team developed a complex intervention to support the transformation of regional primary care into a more integrated model. This intervention, named “COMPAS” (Collectif pour les Meilleures Pratiques et I’Amélioration des Soins et services en médecine de famille), is founded on a comprehensive approach to performance measurement. A focus on population-based assessment of care and action planning is used to facilitate the development of interprofessional and interorganizational collaboration, in order to engage primary care professionals in quality improvement. The objectives of this study were to explain explicitly the theory underlying this intervention, to describe its components in detail and to assess the intervention’s feasibility, acceptability and preliminary outcomes.

## Materials and methods

A program impact theory-driven evaluation approach was used. Multiple sources of information were examined to make explicit the theory underlying the intervention: 1) a literature review and a review of documents describing the program’s development; 2) regular attendance at the project’s committee meetings; 3) direct observation of the workshops; 4) interviews of workshop participants; and 5) focus groups with workshop facilitators. Qualitative data collected were analyzed using thematic analysis. Information on developed actions plans were also collected to document preliminary outcomes of the intervention.

## Results

The theoretical basis of the intervention was found to be work motivation theory. Five themes describing the workshop objectives emerged from the qualitative analysis of the interviews conducted with the workshop participants. These five themes were the importance of: 1) adopting a regional perspective, 2) reflecting, 3) recognizing gaps between practice and guidelines, 4) collaborating, and 5) identifying possible practice improvements. The intervention was offered in nine settings and 22 small groups of primary care professionals developed an action plan. The action plans targeted mainly secondary prevention and were congruent with recommendations from guidelines. The most often identified priorities were improvement of systematic clientele follow-up, greater pharmacist participation and support to improve diabetes self-management. However, the lack of time, resources, leadership, or organizational support was a barrier to the implementation of some action plans.

## Conclusions

Our results confirmed that the intervention enabled professionals to target priorities for practice improvements and to develop action plans that promote improved interprofessional collaboration.

